# Vestibular Morphological Alterations in Adolescent Idiopathic Scoliosis: A Systematic Review of Observational Studies

**DOI:** 10.3390/children10010035

**Published:** 2022-12-24

**Authors:** Irene Cortés-Pérez, Lourdes Salamanca-Montilla, Francisca Gámiz-Bermúdez, Esteban Obrero-Gaitán, Alfonso Javier Ibáñez-Vera, Rafael Lomas-Vega

**Affiliations:** 1 Department of Nursing, Physiotherapy and Medicine, University of Almeria, Road Sacramento s/n, 04120 Almeria, Spain; 2 Department of Health Sciences, University of Jaen, Campus las Lagunillas, 23071 Jaen, Spain; 3 Unidad de Gestión Clínica Adra, Distrito Sanitario Poniente de Almería, Avenida Picasso, 1, 04770 Adra, Spain

**Keywords:** scoliosis, adolescent idiopathic scoliosis, vestibular disorders, vestibular system, vestibular apparatus

## Abstract

Adolescent idiopathic scoliosis (AIS) is the most frequent pediatric spinal deformity. Its treatment still shows limited results due to the existent lack of knowledge regarding etiopathogenesis. Thus, the purpose of the study is to check the existence of vestibular morphological alterations among idiopathic scoliosis patients. To meet the objective, we performed this systematic review searching studies in PubMed Medline, SCOPUS, Web of Science, CINAHL Complete and SciELO until 15 September 2022. Articles that analyzed the morphology of the vestibular apparatus were selected, comparing subjects with AIS versus healthy subjects. Variables were selected that measured the orientation of the channels as well as the general conformation of the vestibular apparatus. One hundred and eighty-five records were retrieved in the preliminary searches, of which five studies were finally included, providing data from 154 participants (83 cases and 71 healthy controls) with a mean age 16.07 ± 2.48 years old. Two studies conclude that the superior and lateral semicircular canals are longer and thinner in patients with AIS. One study concluded that the measure between centers of superior and lateral canals and the angle whose vertex is placed the center of posterior canal were significantly shorter in subjects with AIS than in healthy controls in the left-side of vestibular apparatus. Two studies found an asymmetry in the verticality of the lateral canals on both sides in subjects with AIS, although it is not clear whether the left canal is in a more horizontal or vertical position. Patients with AIS seem to present morphological asymmetries of the vestibular apparatus, fundamentally on the left side. These anomalies seem to correlate with the location of the curve but not with its laterality or severity.

## 1. Introduction

Adolescent Idiopathic Scoliosis (AIS) is the three-dimensional spine deformity of over 10 degrees (measured with Cobb method) in the anterior-posterior dimensional plane that usually appears in adolescents along their development [1]. Around 80% of all spinal deformities in pediatric patients are cases of AIS [2] and a general prevalence between 2 and 3% in adolescents [3]. The overall incidence is 1.44 times higher in women [4]. Patients with AIS present a large variety of physical symptoms, such as movement disorders, reduced strength, spinal pain [5] or difficulty to breathing due to the progression of the pathologic curvature [6,7]. In addition to physical impairments, psychological impairments due to surgical therapy can produce anxiety, depression, or pain catastrophizing, reducing personal autonomy and quality of life [8]. 

This approach is challenging since there is a lack of knowledge regarding the etiopathogenesis, which prevents addressing the cause. Bone and tonic-muscular alterations have classically been studied as the etiology, being analyzed changes associated with growth peaks, with hormones and puberty. Other authors have considered vitamin D deficiency [9] and genetic transmission [10], although genetic analysis points to a high heterogeneity of etiology [10]. However, developments in neuroscience opened new paths based on postural control mechanisms. The vestibular function in subjects with AIS in comparison with healthy subjects has been studied with different measures. On the one hand, Obrero-Gaitán et al. confirmed that patients with idiopathic scoliosis present alterations in vestibular function using the subjective visual vertical test (SVV) [11]. On the other hand, Le Berre et al. [12] assessed the vestibular function of these subjects, compared to healthy controls, using the subjective postural vertical test (SPV), observing that scoliotic subjects showed SPV alteration compared to healthy subjects. A study conducted on frogs showed that an unilateral labyrinthtomy caused a spinal deviation due to an axial asymmetric muscular tonus [13], demonstrating a relation between vestibular system and spinal curvature. In the same line, de Waele et al. used guinea pigs to assess the effects of selective injuries of otolith receptors on the spinal curvature, observing the apparition of scoliotic curves with rotation to the contralateral side of the lesion [14]. Nonetheless, Zagalaz-Anula et al. [15] showed that postural improvements in the spine curve of patients with AIS caused a worsening in verticality perception of the patients, which entails once again that vestibular system seems to play a crucial role in this disease. A possible misfunctioning of the vertical perception would make AIS patients to feel right in a distorted position [15]. 

Regarding the confirmation or rejection of this hypothesis, the aim of this study is to collect all available scientific evidence to determine the possible existence of vestibular morphological alterations in patients with AIS, in comparison with healthy controls, and establish the possible relationship between both conditions.

## 2. Materials and Methods

### 2.1. Review Design

The Meta-Analysis of Observational Studies in Epidemiology (MOOSE) Group guidelines [16], the Preferred Reporting Items for Systematic Reviews and Meta-Analyses (PRISMA) statement [17], and the Cochrane Handbook for Systematic Reviews of Interventions [18] were used to carry out the analysis and report the findings of this systematic review. The protocol of this systematic review was registered in PROSPERO database (CRD42022366524).

### 2.2. Literature Search and Bibliographical Sources

The literature search was performed in PubMed Medline, Web of Science (WOS), SCOPUS, CINAHL Complete, and SciELO up to 15 September 2022 by two authors (I.C.-P. and A.J.I.-V.), independently. The searches in databases were complemented with searches in other sources, such as in the references of papers previously published, in abstracts or proceedings of congress and in document of experts. To perform the search strategy, we identified two search domains: scoliosis and vestibular system. In accordance to the PubMed Thesaurus for Medline (MeSH) the main terms employed were “scoliosis”, “vestibular system” and “vestibular diseases”. In addition, we used synonymous or entry terms related with these keywords, such as “idiopathic scoliosis”, “adolescent idiopathic scoliosis”, “vestibular apparatus”, “vestibular disorders” or “vestibulopathy”. The Boolean operator “AND” was used to join the two domains and synonymous in each domain were linked with “OR”. In our search, we avoid using publication date, language, and free full-text access restrictions or filters. The process was supervised by a third expertise author (R.L.-V.). Table 1 shows the search strategy used in each database.

### 2.3. Study Selection: Inclusion and Exclusion Criteria

This phase was performed by two authors (I.C.-P. and F.G.-B.), who independently checked by title/abstract all records retrieved from the databases and additional sources. When one of the authors identified a record as susceptible to be included in the qualitative synthesis, this record was examined in detail by two other authors. In addition, a third author (R.L.-V.) supervised this phase and decided if an article can be included in case of discrepancy between the two authors.

The studies included in our systematic review must meet these inclusion criteria: (1) observational studies, such as cross-sectional, cohorts, and cases and controls studies; (2) the sample was composed of adolescents with idiopathic scoliosis; (3) to be compared with healthy subjects; (4) and analyze the anatomy of the vestibular apparatus before any therapy. The exclusion criteria to take into account were: (1) studies carried out in animals; (2) observational studies without comparison group; (3) comparison group integrated by subjects with disease and without disease, at the same time; and (4) studies that did not assess vestibular anatomy.

### 2.4. Data Extraction

Two authors (I.C.-P. and F.G.-B.) independently extracted data from the selected studies using a standardized data collection form designed for this review. The discrepancies between the authors were solved by a third author (R.L.-V.). We extracted data related to authorship, publication date, country, sample size, number of participants in each cases and control groups (cases or patients with AIS, and controls or healthy subjects), age, sex, and time since diagnosis. We compiled data of the variable of interest, the measurement tool employed, and main findings.

### 2.5. Outcomes

Variables were selected that measured the orientation of the channels as well as the general conformation of the vestibular apparatus, the length and thickness of the semicircular canals, as well as the angle that the different canals form with each other.

### 2.6. Methodological Quality Assessment

Two authors (E.O.-G. and L.S.-M.) independently assessed the methodological quality of the studies included using the Newcastle-Ottawa Scale (NOS) [19]. This scale assesses three domains: selection, comparability, and ascertainment of exposure in cohorts and case and controls studies. The score range varies from 0 stars (poor methodological quality) to 9 stars (large methodological quality) [20]. The methodological quality of the observational studies can be classified as low (1–3 stars), medium (4–6 stars), and large (7–9 stars) [21].

## 3. Results

### 3.1. Study Selection

**Figure 1:**
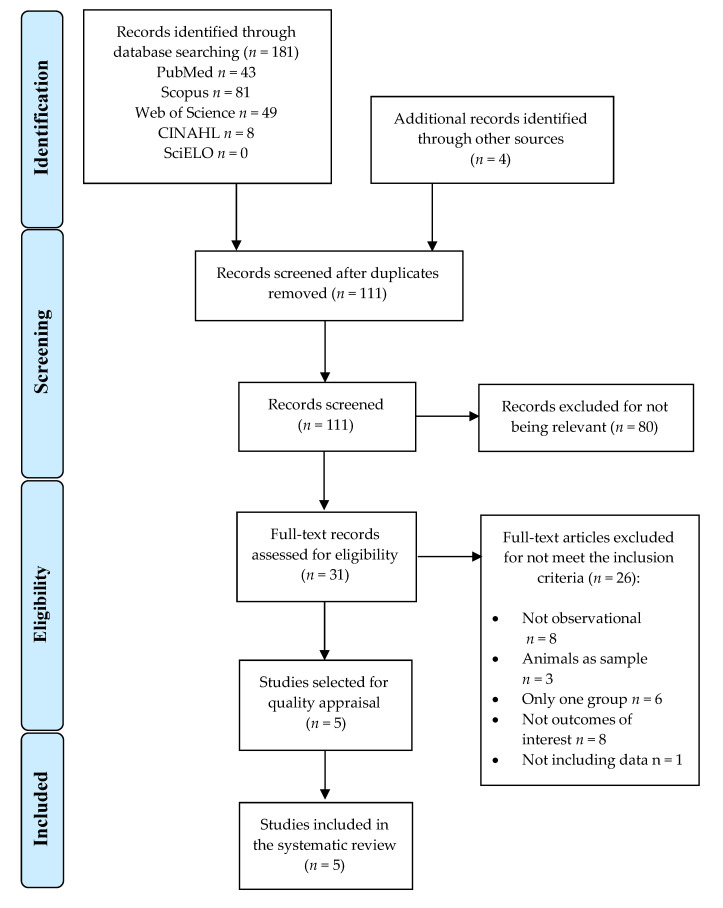
PRISMA flow diagram.

One hundred and eighty-five records were retrieved in the preliminary searches (*n* = 181 from databases and *n* = 4 from other sources). After deleting 74 duplicates, 111 references were screened by title/abstract. Eighty studies were excluded for not being relevant and 26 for not meeting the inclusion criteria. Finally, 5 studies were included in the present systematic review [22,23,24,25]. PRISMA flow chart (Figure 1) shows the study selection process.

### 3.2. Characteristics of Studies Included in the Review

The five studies were carried out in United States of America [22,24], China [23,26], and France [25] between 2010 and 2020. The studies included provided data from 154 participants with a mean age of 16.07 ± 2.48 years old (96% females). The case group was comprised of 83 subjects with AIS (97% females) with a mean age of 14.95 ± 0.39 years and a mean Coob’s angle of 32.82 ± 10.72 degrees. The control group was formed by 71 healthy participants (95% females) with a mean age of 17.93 ± 3.61 years. All studies included in this review received external funding. Table 2 shows the main characteristics of the studies included.

### 3.3. Methodological Quality Assessment

The mean methodological quality of the included studies assessed with NOS was moderate (mean score of 5 ± 1.41 stars). Four studies [22,23,24] (80%) presented moderate quality and one study [25] (20%) showed high quality. Table 3 shows the NOS score for each study included.

### 3.4. Main Findings in Studies Included

Of the five works selected, the two oldest by Zeng et al. [24] and Xin et al. [23], used a similar methodology based on magnetic resonance image to establish the length and width of the semicircular canals, focusing on those on the left side. The two studies used similar samples of 15 and 11 patients with thoracic AIS, obtaining significant differences versus the control patients in terms of length and width. Both works conclude that the superior and lateral semicircular canals are longer and thinner in patients with AIS (Table 2).

The work of Shi et al. [26] analyzed the distance and angle between the semicircular canals of the two sides in a sample of 20 right-thoracic AIS girls (7 severe; 8 moderate; 5 mild) and 20 age-matched healthy girls concluding that distance between centers of lateral and superior canals and the angle with vertex at the center of posterior canal were significantly smaller in AIS than in healthy controls in the left-side vestibular system (Table 2).

Finally, the works by Hitier et al. [25] and Carry et al. [22] used a similar methodology to analyze the relationship of the semicircular canals with respect to the body midline and the horizontal plane, reaching opposite conclusions (Table 2). While Hitier et al. found that the left lateral SCC is more vertical in scoliosis [25], Carry PM et al. found that among subjects in the AIS, the left lateral SCC tended to be oriented in a more horizontal position than in healthy subjects [22].

## 4. Discussion

This review aimed to detect the best scientific evidence on the relationship between morphological alterations of the vestibular system and adolescent idiopathic scoliosis. The search located five works that, using very novel analysis methodology of magnetic resonance image, have tried to quantify the morphological asymmetries of the vestibular systems of patients with AIS in comparison with healthy subjects. Given the special anatomical conformation as well as the small size of the vestibular apparatus and its components, finding measurable differences between the two sides of a subject or between patients and healthy controls is a real challenge. In general, studies have focused on the analysis of the vestibular apparatus on the left side [24] or have found alterations on this side [22,25,26], which seems to be the one that shows the clearest anatomical differences between subjects and controls. This could explain the greater frequency of right curves, but it does not explain the great diversity of curve types when the factors of laterality, location, and severity are taken into account. The main alterations found in AIS subjects were thinner lateral and superior canals [24], longer lateral and superior canal [23], different angle of the left posterior canal [26], and smaller/higher angle formed by both lateral SCCs [22,25]. Studies that found morphological abnormalities of the vestibular apparatus have not always been able to identify a correlation between these anatomical findings and abnormalities of vestibular function [25]. However, it has been observed that the intercanal angle (IA) could differentiate between thoracolumbar scoliosis, in which the IA would be smaller compared to lumbar scoliosis. Thoracolumbar scoliosis also showed a more marked asymmetry in the location of lateral SCCs with a right canal located more laterally than the left compared with dorsal or lumbar curvatures. No correlation could be identified between the morphology of the semicircular canals and laterality, the severity of the curve or the age of the patients.

Of the studies included in this review, only one [25] analyzed the presence of functional anomalies of the vestibular system associated with morphological alterations. In this study, it was found that paresis of the semicircular canal presented a certain correlation with the morphological parameters such as the intercanal angle, the “vertical lateral canal frontal angle right” (VLFR) formed between the right lateral SCC and the vertical axis (defined by the sagittal plane), and the “vertical lateral canal frontal angle left” (VLFL) formed between the left lateral SCC and the vertical axis, as well as the lateral distance and the anterior distance of the left SCC [25]. However, functional alterations failed to differentiate between IS patients and healthy controls. In other words, the functional alterations that accompany the vestibular morphological alterations that have been studied in the scientific literature do not seem to mediate the presence of IS. However, another study, by examining postural and muscle responses to binaural bipolar galvanic vestibular stimulation (GVS) of randomly alternating polarity, has found a vestibular imbalance in adolescents with idiopathic scoliosis that is compensated by somatosensory, load-related afferent feedback from the lower limbs during the latter part of the response [27]. These findings could indicate that beyond the functional impairment at the vestibular level, the dysfunction would be caused by the way in which vestibular signals are integrated with somatosensory information to produce postural responses.

The interest in the relationship between the vestibular system and the AIS is recurrent and stems from findings referring to possible functional alterations of the postural control system with this structural anomaly of the spine [27,28]. In this line, Lambert et al. (2009) observed that an unilateral labyrinthtomy produced on frogs caused spinal deviation due to an axial asymmetric muscular tonus [13]. Similar observation was made by Wilczynki in 2021, determining in humans that erector spinae muscles tone in AIS subjects presented a different behavior in the convex/concave side of the curve [29]. Thus, an alteration in vestibular system and the neural descending pathways added to the lack of other proprioceptive inputs in aquatic environment and resulted in the asymmetric development of spine in frogs, which could be compared with the environment during human gestation in uterus [13,30]. Similar observation was determined by de Waele et al. in mammals, as the lesion of selective otoloyth receptors in guinea pigs caused them a spinal deviation toward the opposite side of the injury [14].

The assessment of vestibular system function has been a challenge, despite of the existing several tools and validated procedures. One of the accepted measures for vestibular function is the perception of visual verticality [31]. However, the perception of verticality does not seem to be altered in subjects with AIS [11], which could be interpreted as the perception of visual verticality is in balance with spinal curvature, since some study has found that subjects subjected to curve treatment could worsen the perception of visual verticality [15].

A recent review concluded that it seems unlikely that an isolated vestibular disorder could trigger structural scoliosis [32], although pathologies of the vestibular system can certainly occur in the multifactorial genesis of idiopathic scoliosis. In this sense, the work by Hitier M et al. found that an intercanal angle of less than or equal to 170° was 100% specific for scoliosis, but showed a sensitivity of 59%, that is, there are 41% of scoliotic subjects showing an intercanal angle that cannot explain their scoliosis compared to similar angle in subjects without scoliosis, which shows the multifactorial nature of the problem.

In relation to other morphological abnormalities that could be the cause of AIS, some studies have found an alteration in the thickness of the vascular layer of the eye in patients with spinal curvature [33]. This finding is of interest for our work because it will affect the postural control disorder due to the great importance of vision for postural balance and the fact that this alteration of the ocular structure has been related to aneisiconia that can cause asymmetry in the size of the images that are perceived with each eye [34]. We do not know yet if a relationship exists between ocular disorders and asymmetry of the vestibular apparatus, which needs to be studied in future with independent causal factors.

Other works have related AIS with morphological or functional alterations of the stomatognathic apparatus [35]. These findings usually present the same problem of interpretation that could be applied to the relationships between AIS and vestibular anomalies, that is, we do not know which factor is the cause and which is the consequence. However, some classic works [36] found that congenital orthodontic anomalies that are present from birth are related to a higher probability of developing scoliosis during adolescence. Animal models have also provided evidence that occlusion manipulation in rats can cause spinal structural deviations that may be reversible by reversing the effect of occlusion manipulation [37].

Although it appears that at least a subgroup of patients with AIS may have morphological abnormalities of the vestibular apparatus, published studies have not been able to discern whether vestibular asymmetry may be the cause or the result of the skeletal torsion that occurs in AIS. On the other hand, further evidence is required with large samples that could establish patterns of vestibular deformity and correlate them with the type, location, and severity of the curve. The other factors related to the appearance of AIS and that are present in patients with spinal deformity but with correct spinal morphology remain to be identified.

Concerning AIS approach, our findings suggest that morphological abnormalities of the vestibular apparatus (independently of the type) could be considered among others as an etiological factor. For this reason, further research is needed to provide successful approaches for this subgroup of patients.

Despite findings presented in this systematic review are interesting for neuro-otology field, some limitations must be taken into account. At first, the low number of studies included can reduce the generalization and robustness of our findings. Second, NOS assessment advertises a possible risk of selection bias. Third, these findings have not been corroborated with statistical analysis, and publication bias cannot be assessed.

## 5. Conclusions

AIS patients seems to present morphological alterations in the vestibular apparatus that could be associated with the etiology or development of the spinal deformity. These alterations could affect the length and thickness of the anterior and lateral semicircular canals on the left side as well as the angulation of the lateral canal relative to the transverse plane, although it is not clear whether the problem is an increase or decrease in verticality of the canals. It has not yet been possible to establish whether the vestibular structural asymmetry is the cause or the consequence of the spinal deformity, nor has a relationship been established between the vestibular deformity with the general characteristics of the curve and the functional abnormalities found in the subjects with AIS.

## Figures and Tables

**Table 1:** Search strategy used in each database.

Databases	Search Strategy
PubMed Medline	(scoliosis[mh] OR scoliosis[tiab] OR idiopathic scoliosis[tiab] OR adolescent idiopathic scoliosis[tiab] OR congenital scoliosis[tiab] OR neuromuscular scoliosis[tiab]) AND (vestibular system[mh] OR vestibular system[tiab] OR vestibular apparatus[tiab] OR vestibular anatomy[tiab] OR semicircular ducts[mh] OR semicircular duct*[tiab] OR vestibule, labyrinth[mh] OR vestibule, labyrinth[tiab] OR saccule[tiab] OR utricle[tiab] OR otolithic* organ*[tiab] OR vestibular nerve[mh] OR vestibular nerve[tiab] OR vestibulo-cochlear nerve[tiab] OR vestibular disorders[mh] OR vestibular disorders[tiab] OR vestibular disease*[tiab] OR vestibular dysfunction*[tiab])
SCOPUS	TITLE-ABS-KEY (“scoliosis” OR “idiopathic scoliosis” OR “congenital scoliosis” OR “adolescent idiopathic scoliosis”) AND TITLE-ABS-KEY (“vestibular system” OR “vestibular anatomy” OR “vestibular apparatus” OR “vestibular labyrinths” OR “saccule” OR “utricle” OR “vestibular nerve” OR “vestibular diseases” OR “vestibular disorders” OR “vestibular dysfunctions”)
Web of Science	TOPIC (*scoliosis* OR *idiopathic scoliosis* OR *congenital scoliosis* OR *adolescent idiopathic scoliosis*) AND TOPIC (*vestibular system* OR *vestibular anatomy* OR *vestibular apparatus* OR *vestibular labyrinths* OR *saccule* OR *utricle* OR *vestibular nerve* OR *vestibular diseases* OR *vestibular disorders* OR *vestibular dysfunctions*)
CINAHL Complete	AB (scoliosis OR idiopathic scoliosis OR adolescent idiopathic scoliosis) AND AB (vestibular system OR vestibular apparatus OR vestibular anatomy OR vestibular diseases)
SciELO	Scoliosis AND (vestibular system OR vestibular diseases)

* is used to search the root of a word

**Table 2:** Main results of the included studies.

Study	Sample	Focus	Variables	Results	Conclusions
Zeng, W., et al., 2010 (United States of America) [24] Setting: Not reported Funding: Yes	-15 girls with right-thoracic AIS (mean age 15 Years old; mean Cobb’s angle 27.27 degrees) -12 age-matched healthy girls	Left side vestibular system	-Length of the semicircular canals (MRI) -Thickness of the semicircular canals (MRI)	-Lateral andsuperior canals are generally thinner for AIS subjects -The lateral canal are significantly different between AIS and Controls	The left semicircular canals are longer and thinner in girls with AIS. The lateral semicircular canal is different in girls with scoliosis.
Xin, S.Q., et al., 2011 (China) [23] Setting: Not reported Funding: Yes.	-11 girls with right-thoracic AIS (mean age 15 years old with variance 1.7 yearsold); (mean Cobb’s angle 27.27 degrees with variance 15.62 degrees) -11 age-matched healthy girls.	Vestibular system. Side no declared.	-Length of the semicircular canals (MRI) -Thickness of the semicircular canals (MRI)	-Lateral and superior canals are generally longer for AIS subjects. -Lateral and superior canals are significantly different between groups	The shape in the lateral and superior canals tends to be significantly different between the two groups.
Shi, L., et al., 2011 (China) [26] Setting: Scoliosis Clinic of Prince of Wales Hospital (Hong Kong) Funding: Yes	-20 right-thoracic AIS girls with a mean age of 14.7 years old and mean Coob’s angle of 32.6) -20 age-matched healthy girls (mean age of 15.1 years old)	Left and right side of vestibular system.	Distance and angle between the semicircular canals (MRI)	-On the left side, the distance between the lateral and superior channels is different between AIS and controls. -On the left side, the posterior canal angle differs between AIS and controls	Distance between centers of lateral and superior canals and the angle with vertex at the center of posterior canal were significantly smaller in AIS than in healthy controls in the left-side vestibular system.
Hitier, M., et al., 2015 (France) [25] Setting: School environment Funding: Yes	-17 AIS with 15.47 ± 1.84 years old and a mean Coob’s angle of 26.7 ± 8.3 degrees. -9 healthy controls with 16.7 ± 1.5 years old.	Angular relationships of the semicircular canals on both sides.	Lateral semicircular canal orientation and the three semicircular canal positions relative to the midline (MRI)	-Both lateral SCCs formed a smaller angle together in the scoliosis group -An intercanal angle of less than or equal to 170° was 100% specific for scoliosis, with a sensitivity of 59%	Left lateral SCC is more vertical in scoliosis Left lateral SCC and posterior SCC are further from the midline in scoliosis
Carry, P.M., et al., 2020 (United States of America) [22] Setting: Research Institute at Children’s Hospital (Colorado) Funding: Yes	-20 females with AIS (14.61.9 years old) and mean Coob’s angle of 49.83 ± 4.61 degrees. -19 healthy controls (22 ± 7.8 years old)	Horizontal distance from the lateral and posterior SCCs to the anatomical midline	Orientation of right versus left lateral SCC (MRI)	-The average differencein the lateral SCC angle between R and L sides was higher in the AISgroup compared to the control group -The side-to-sidedifference in the lateral SCC angle was higher in the AIS relativeto the control group	Compared to the right side, AIS patients have a more horizontally oriented left lateral semicircular canal than control subjects.

MRI: measured on magnetic resonance image; SCC: semi-circular canal AIS: adolescent idiopathic scoliosis.

**Table 3:** NOS score of the studies included in the review.

Study	S1	S2	S3	S4	C	E1	E2	E3	Score	Quality
Zeng, W., et al., 2010 [24]	-	-	-	*	**	-	-	*	4/9	Moderate
Xin, S.Q., et al., 2011 [23]	-	-	-	*	**	-	-	*	4/9	Moderate
Shi, L., et al., 2011 [26]	*	-	-	*	**	-	*	*	6/9	Moderate
Hitier, M., et al., 2015 [25]	*	*	*	*	**	-	*	-	7/9	High
Carry, P.M., et al., 2020 [22]	-	-	-	*	**	-	-	*	4/9	Moderate

Each study can be awarded a maximum of one star for each numbered item within the Selection (S) and Exposure (E) categories. A maximum of two stars can be given for Comparability (C). S1, adequate case definition; S2, representativeness of the cases; S3, selection of controls; S4, definition of controls; C1, comparability of cases and controls; E1, ascertainment of exposure; E2, same method of ascertainment for cases and controls; E3, non-response rate; “*”, 1 Star or 1 point; “**”, 2 stars or 2 points; “-“ no stars or 0 points.

## Data Availability

Not applicable.
